# Barriers and facilitators to the implementation of a community-based hypertension improvement project in Ghana: a qualitative study of ComHIP

**DOI:** 10.1186/s12913-019-4774-x

**Published:** 2020-01-30

**Authors:** Alma J. Adler, Amos K. Laar, Agnes M. Kotoh, Helena Legido-Quigley, Pablo Perel, Peter Lamptey, Isabelle L. Lange

**Affiliations:** 1 Department of Global Health and Social Medicine, Harvard Medical School Boston, Boston, MA USA; 2 Department of Non-communicable Disease Epidemiology, London School of Hygiene & Tropical Medicine, Keppel St, London, WC1E 7HT UK; 3 Department of Population, Family, & Reproductive Health, School of Public Health, University of Ghana, LG 13, Legon, Accra Ghana; 4 FHI360, Washington DC, WA USA; 5 Department of Infectious Disease Epidemiology, London School of Hygiene & Tropical Medicine, Keppel St, London, WC1E 7HT UK

**Keywords:** Hypertension, Implementation research, Facilitators, Ghana, Community-based, Qualitative research

## Abstract

**Background:**

Globally, hypertension is a leading cause of cardiovascular disease and mortality, with the majority of deaths occurring in low- and middle-income countries. Because the burden of hypertension is increasing in low resource settings with restricted infrastructure, it is imperative that new models for hypertension care are realised. One such model is the Community-based Hypertension Improvement Project (ComHIP) which employs a community-based method of task-shifting for managing hypertension. This study is a qualitative analysis of the barriers and facilitators of the main components of ComHIP.

**Methods:**

We purposively selected 55 informants for semi-structured interviews or focus group discussions, which were carried out bythree trained local researchers in Krobo, Twi or English. Informants included patients enrolled in ComHIP, health care providers and Licensed Chemical Sellers trained by ComHIP, and Ghana Health Service employees. Data were analysed using a multi-step thematic analysis.

**Results:**

While results of the effectiveness of the intervention are pending, overall, patients and nurses reported positive experiences within ComHIP, and found that it helped enable them to manage their hypertension. Healthcare providers appreciated the additional training, but had some gaps in their knowledge. Ghana Health Service employees were cautiously optimistic about the programme, but expressed some worries about the sustainability of the programme. Many informants expressed concerns over the inability of community nurses and workers to dispense anti-hypertensives, due to legal restrictions.

**Conclusions:**

The WHO recommends task-sharing as a technique for managing chronic conditions such as hypertension in resource constrained settings. ComHIP presents an example of a task-sharing programme with a high level of acceptability to all participants. Going forward, we recommend greater levels of communication and dialogue to allow community-based health workers to be allowed to dispense anti-hypertensives.

## Background

Globally, raised blood pressure is a leading cause of cardiovascular disease (CVD) with nearly 10.4 million annual deaths [1]. The level of blood pressure where treatments show reduction in clinical events in randomised trials is generally accepted as ≥140 systolic mmHg (SBP) or ≥ 90 diastolic mmHg (DBP). This level is termed ‘hypertension’ [2]. For individuals diagnosed with hypertension, evidence shows that lowering blood pressure with accessible drugs reduces the risk of subsequent cardiovascular events, with an estimated 35–40% reduction in the risk of stroke and a 20–25% reduction in the risk of myocardial infarction and heart failure [3–5]. The hypertension prevalence in many low- and middle-income countries (LMICs) is as high as or higher than in high-income countries [6–8]. Between 32 and 50% of adults are estimated to be hypertensive with an increasing burden in sub-Saharan Africa [6].

The Prospective Urban Rural Epidemiology (PURE) study showed that despite high levels of hypertension worldwide, awareness of hypertension is low, with only 34% of Africans aware of their hypertension status, 31.3% receiving treatment and 6.5% with it under control [9]. Like other LMICs, Ghana is facing an increasing burden of non-communicable diseases (NCDs), with hypertension at the forefront. Prevalence of hypertension in Ghana ranges from 24 to 28% among women and 20 to 33% among men [6, 10–12]. Traditionally, the Ghanaian healthcare system has focused on maternal and child health and communicable diseases. Ghana’s first ever social protection initiative that addressed NCDs was the National Health Insurance Scheme (NHIS), established by the National Health Insurance Act, 2003 (Act 650). Its aim was to increase access to quality health care services for all residents and to replace out-of-pocket payments referred to as ‘cash-and-carry’. The benefit package covers about 95% of diseases in Ghana including hypertension [13], however as of 2016, only about 40% of the total Ghanaian population (estimated 26.9 million) was enrolled in the NHIS with valid membership cards [14]. Currently, the NHIS is available countrywide through both public and accredited private health facilities, including chemical stores (licensed pharmacies) and laboratories [13].

Given the rising burden, it is imperative to explore innovative methods of increasing access to NCD prevention and care services; particularly improving access to hypertension care. One programme being implemented in the eastern region of Ghana is the Community-based Hypertension Improvement Project (ComHIP). The project tests a community-based model which engages the private sector and utilises information and communication technologies (ICTs) in the Manya Krobo district. Prior to ComHIP, despite a heavy burden, there were a limited number of NCD programmes in the Manya Krobo, including the Ghana Health Service (GHS) standard of practice (targeted screening and mass communication) where hypertension management is largely restricted to hospitals. To fill the knowledge gap on the implementation of hypertension interventions, this paper presents a qualitative investigation into the acceptability of the ComHIP programme among its users and the barriers and facilitators to its uptake.

### The community-based hypertension improvement project

ComHIP is a public-private partnership involving GHS, FHI360 and the Novartis Foundation, supported by the University of Ghana School of Public Health and the London School of Hygiene & Tropical Medicine. ComHIP is aimed at addressing knowledge and management of hypertension through a community-based model using task-sharing, community mobilisation, and ICT to increase knowledge of hypertension and provide access to anti-hypertensive drugs. Designed as implementation research, ComHIP comprises implementation and evaluation (done prior, during and post intervention). The six components of ComHIP are:

1. Community-based education on CVD risk factors and healthy lifestyles using knowledge creation activities including local FM broadcasts and traditional community sensitisation activities such as community durbars (form of organised community meeting with all key community stakeholders) and beating the *gong gong* (a traditional instrument used by community announcers to deliver important messages from chiefs, their elders, and government and non-governmental agencies to residents)*.*
2. Community-based screening and monitoring of blood pressure by Licensed Chemical Sellers (LCS) trained by FHI360
3. Management of hypertensive individuals by CVD nurses for confirmation of hypertension diagnosis, management, and follow-up. CVD nurses are nurses who carry out primary care whilst posted in communities, and who have been trained by FHI360.
4. Telemedicine consultation by CVD nurses or ComHIP trained physicians and referral of high risk (defined as grade three hypertension, or grade two hypertension with target organ damage or two or more risk factors) patients to specialists.
5. ICT messages for healthy lifestyles, treatment adherence and refill reminders that were routinely sent to enrolled patients
6. A cloud-based health records system linked to Short Message Service (SMS) messaging (CommCare)

All data collection tools were built on the CommCare communication platform, and loaded onto case management mobile devices. Once an individual was confirmed hypertensive in CommCare, an algorithm advised the CVD nurse to enrol the patient into ComHIP. The enrolment form creates a case and generates a unique identity code for the client. This allows for all recorded client information – such as referral, prescription, follow-up and dispensing forms to be linked. Nurses, LCS, physician assistants and ComHIP physicians were provided Samsung tablets to facilitate their work.

Of particular note, due to financial complications, CommCare was non-operational from approximately April 12th through July 27th 2017, which meant that records were not linked and messages were not sent during this time period.

This paper presents results of an assessment of the six main components of ComHIP, focusing on the barriers and facilitators of each component using qualitative methods.

## Methods

### Study design and area

This qualitative study was conducted in the Lower Manya Krobo District in the Eastern Region of Ghana to assess the barriers and facilitators associated with adhering to the ComHIP programme. This is a peri-urban district with a population of 89,246 with approximately 84% living in urban centres [15]. The district is zoned into six sub-districts and has two public hospitals, and four public health centres, 14 community-based health planning and services (CHPS) zones and approximately 75 LCSs. The CHPS zones serve as primary contact points for health care referring patients to hospitals.

### Informants and data collection

A total of 55 informants were purposively selected. Gender, age, district and socio-economic status were considered to ensure they fairly represented each category of study participant (Table 1). Thematic saturation was established when all researchers discussed and agreed that a diversity of responses had been obtained and no new themes were emerging from the data. Health care providers were selected to ensure representativeness of centres and LCSs in the studied communities, they included seven nurses, one clinician, one physician’s assistant and a pharmacist. Policy makers were selected based on the relevance of their position within NCDs. They included the National Programme Manager of NCDs, the Greater Accra Regional Director of Health Services, the Director of Policy, Planning, Monitoring and Evaluation of the Ghana Health Service. Further information and guides can be found in a previous publication [16].

**Table 1:** Categories of informants interviewed

Informants	In-depth interviews	FGD	Total
Patients	15	16	31
Healthcare professionals	10	–	10
Licensed chemical sellers	–	7	7
Policy makers	7	–	7
Total	32	23	55

Three trained researchers fluent in English and Krobo or Twi carried out face-to-face semi-structured interviews and focus group discussions (FGD). Two qualified researchers conducted a one-week training session to ensure that interviewers understood the objectives of the study and the questions. The research team was trained to respond to any sensitive situations or signs of distress with appropriate wording, supportive statements and avoidance of excessive probing. A pre-test was done in a different district to standardise the interview guides. Based on participant’s preference, interviews and FGDs were conducted in English, Krobo or Twi and audio recorded.

We interviewed 15 patients enrolled in ComHIP. Interviews were conducted privately, either at health centres or at participants’ homes, and ranged from 30 to 60 min. The interview topics for hypertensive patients included: a) knowledge of hypertension and its diagnosis, b) how treatment and prevention of hypertension is provided, c) experiences when seeking care and recommendations for improvement, d) health system barriers and facilitators to accessing care for hypertension, and e) factors influencing adherence. In intervention studies, changes in individual behaviours and institutional practices can be measured using surveys, but miss the critical information about participants’ perspectives and contexts gathered through qualitative methods that can help explain the causal relationship.

We interviewed ten healthcare professionals: seven CVD nurses, one pharmacist, one physician assistant, and one physician. The topics for health care professionals included: a) experience and tasks, b) how treatment and care is provided and coordinated, c) how treatment has changed with the programme, d) their relationships with patients, and e) areas for improvement at the health systems level.

The seven interviews with policy-makers were tailored to their expertise and position. The interviews focused on understanding NCD programmes, the key barriers in the implementation of hypertension programmes, key health systems-level facilitators, who the key actors are, aspects that need improvement, and the commitment to scaling up the ComHIP programme.

We performed two FGDs (one male, one female) with a total of 16 patients to explore experiences of care in ComHIP. The lively group interaction in the FGDs generated a broad range of opinions on the issues mentioned above. We also held one FGD with seven LCSs to discuss their experiences with the programme and opinions on scalability.

### Data analysis

The interviews were audio recorded and transcribed verbatim. The transcriptions were translated from their respective languages to English by expert native speakers in English and Krobo or Twi, and wherever possible by the individual who conducted the interview.

The data were then analysed using thematic content analysis techniques employing two steps. After establishing a-priori themes created from the intervention questions, we manually coded transcripts based on these and other themes that emerged through the fieldwork and analysis process. From this coding we looked for the links among themes and identified overarching concepts. We then held a follow-up meeting between all researchers and research assistants to discuss major themes and issues that were not clear in the transcriptions.

## Results

We have organised the results along the six ComHIP implementation components: community-based education by CVD nurses; screening by LCS; community-based diagnosis by CVD nurses; telemedicine and referral; use of ICT technology; and the CommCare platform. We also include a section on participants’ overall experiences in the programme.

### Programme components

#### Community-based education on risk factors and healthy lifestyle

Patients were invited to enrol in the programme if they were diagnosed with hypertension (SBP ≥140, or DBP ≥90 based on three readings) or were currently taking anti-hypertensives. As part of the programme, all patients received community-based education on CVD risk factors and healthy lifestyles provided by CVD nurses and LCS. The community-based education on hypertension, hypertension risk factors, and risk reduction made use of various knowledge/awareness creation activities using local FM broadcasts. Health education was provided during face-to-face meetings and within the community at prearranged meeting sites. Community sensitization activities made use of popular local techniques, including durbars and *gong gong* beating. These general actions were followed by education initiatives tailored to enrolled patients.

Patients in the programme told the interviewers that through ComHIP they learned the value of monitoring their BP. Some stated that before the programme they had no knowledge about the disease, or thought it was not a relevant disease until they were much older, but now realised the importance of looking after their BP at any age. There was a sense among participants that hypertension was a severe disease that could kill or immobilise you without warning. As one man stated: “*Actually I knew I had this before the onset of this programme but what this programme has done in my life is that it has created this sense of urgency to take my BP more seriously. That is the change I have seen with this programme*.” (Male participant in a focus group) Another participant explicitly stated that without this programme he felt that he could have died due to ignorance of hypertension.

All participants discussed how ComHIP had impacted positively on their lives, and how they had benefited from the health education. When prompted, most participants said that the programme caused them to change their diet, understand the need to exercise and to cut down on alcohol intake. We were not able to confirm the modification of behaviours, but from participant accounts it appeared that the project brought into their consciousness a sense of the negative impact of hypertension.

Nurses reported that while ComHIP has been successful in providing education and raising hypertension awareness, more health education should be available to patients on other topics by the GHS, particularly regarding prevention, healthy habits, side effects, and adherence. One CVD nurse said:

*When we educate them on drinking, their diets, and exercise, I think we will not get many new cases. Most of the women are hard working so with exercise it’s not a problem but the way they eat, their habit of eating … they say: I’m tired. ‘I work; I don’t know when I will die.’ Therefore chofi come, indomie add, egg come, sausage come … that is the issue. So when more education is done on the eating, hypertension will be far, far from them.* (Nurse interview 1)

Nurses also emphasised the importance of counselling to ensure adherence. The following quote illustrates how nurses educate patients to take their drugs every day, even after measured improvements:

*You counsel the client on [adherence] based on lifestyle and then you teach them that it is important that they take their drugs every day. What they normally do is after taking the drugs for some time and the BP becomes normal, they stop taking the drugs. They feel they are okay but you educate them, you counsel them, you give them the advantages and the disadvantages of that, the reasons why they shouldn’t stop taking the drug.* (Nurse interview 7)

A physician assistant also spoke of some of the real-life challenges in providing health education: *“They are above 40 and most of them are already obese. How can you ask this woman to exercise? It is difficult to prescribe exercise for such a person; access to maybe the gym... / … Ah, it is easier said than done. These are some of the challenges.*”

#### Community-based hypertension screening by licensed chemical sellers

LCS work out of local shops and were trained by FHI360 to screen and refer patients for hypertensive care. Per the Ghana Pharmacy Act - 1994 (ACT 489), LCS are registered local service providers who are assessed and deemed fit by the Pharmacy Council to retail restricted drugs other than Class A or B drugs. Originally, ComHIP had planned for permission from GHS to allow LCS to prescribe anti-hypertensives, but this was not allowed. After training, FHI360 provided LCS tablets and Omron BP monitors for customer screening, but provided no financial incentives, assuming that screening would increase traffic to the LCS sufficiently to make the programme profitable for them through the sale of over-the-counter drugs.

Most patients interviewed were not able to differentiate between LCS and community chemists, who are legally allowed to dispense anti-hypertensives and are not trained as part of ComHIP. Patients reported consulting LCS for hypertension screening but not health education. Many patients discussed going to LCS for anti-hypertensives, but as LCS were not licensed to sell them it was unclear if patients were obtaining their medications from illegal stocks at LCS, or if their interactions were with community chemists. Overall, patients reported cordial, pleasant interactions with the LCS.

Some LCS discussed how their role in creating awareness, counselling, and health promotion earned them more respect in the community. They were proud to be the first point of contact for hypertension screening and for their contribution to improving community members’ health and wanted the programme to continue. A LCS stated this explicitly in the FGD:

*Yeah, I think [ComHIP] should continue because when you go deep down to our communities we have so many people there that don’t know anything about BP. Because now when you are at the hospital you see so many people coming around saying my head aches, this place of mine hurts and others, and I think it’s all the symptoms of the BP. So I think we have to continue but the people should be more. If they can employ people so that they can go deep down the roots. We have so many people that don’t know, so, am pleading with you. *(Focus group with LCS)

While they reported some increased revenues from their involvement in ComHIP, LCS expressed dissatisfaction with not being paid by ComHIP for their services. LCS reported some increased traffic from participants coming for screening, but the increased traffic was considerably less than they had originally anticipated, particularly because they were legally unable to dispense medications as originally promised. Of those interviewed, LCS displayed the lowest satisfaction with the programme, particularly regarding their financial revenues. After the FGD was over, several LCS expressed unhappiness with how FHI360 utilised their funds (for example on trainings and hotel accommodation for participants) and would have preferred that they be used for LCS remuneration.

#### Community-based screening, diagnosis, management, and follow up by CVD nurses

The CVD nurse training provided by FHI360 consisted of a six-day course and quarterly meetings where they discussed their experiences and refreshed their skills. Prior to ComHIP, nurses had been trained to varying degrees on CVD during their nursing education, but most had not received any sort of follow-up, and they expressed gratitude for the refresher provided by ComHIP. Our results suggest that before ComHIP, nurses were perceived as existing outside of the community, and did not have much day-to-day interaction with community members.

##### Quality of training provided to health professionals

All health professionals reported that FHI360’s training helped them to perform their new roles. The ICT component of the training was singled out as particularly useful, and others stated that they would have liked more of it. Nurses reported that their knowledge of risk factors, prevention, and treatment of hypertension had improved after the training. Overall, their confidence to advise and talk to their patients increased.

A community staff nurse expressed her experiences in the programme as follows:

*My experience has been very nice. ComHIP has actually helped me a lot because I knew a bit about hypertension, but not that much until the project came in, and since then I have learnt a lot about how to interact with my clients. Though we had previously learnt about this communication and other stuff, this project has given us a wider view about interacting with our clients and how we can educate them.* (Nurse interview 5)

The training appeared to be effective in getting across the roles and responsibilities associated with the programme, but less so the clinical aspects. Many nurses were aware of their tasks, but some appeared not to have a good grasp of the clinical aspects including the existence of clinical guidelines and causes of hypertension. For example, many said that hypertension was caused by stress. So although all nurses found the training to be useful, gaps in their performance suggest that additional training is necessary.

##### Communication and interaction between health-care professionals and patients

Patients reported good mutual communication and respect with the CVD nurses and that they could be “free with” each other (meaning that they felt comfortable talking and joking with them). Their overall feeling was that nurses were teaching them something valuable, and if they listened to them they would become healthy. Similarly, nurses and other health professionals reported being able to communicate with patients and colleagues in part due to the training which allowed them to improve this dynamic. Overall, health professionals did not report many communication barriers except for the linguistic challenges brought up by three health professionals who were not Krobo.

##### Patient-health worker interactions

Patients and nurses both reported that ComHIP had improved their rapport, and that while prior to ComHIP patients did not know the nurses’ names, post-ComHIP they did. Nurses reported having a greater connection with community members and that they now were more popular, appreciated and respected than before the programme. As one CVD nurse stated:

*...and then for me being the only person attending to that particular client … she is now proud she knows you, madam [name], madam [name]. Anytime she gets here, she mentions your name. Gone are the days they don’t even know our names. They come and anybody attends to them. It made me more popular and I even was given gifts through this.* (Nurse interview 1)

Many patients concurred:

*When I was not in this project, I could hardly get close to the nurses even in our clinic here, but when I was enrolled, I now could easily go to them at any time without difficulties as before. I sometimes come to complain about anything that went wrong the previous day to them and I get advice from them.* (Patient interview 5)

Both nurses and patients reported that patients preferred consultations with CVD nurses to physicians. Nurses were perceived as spending more time than doctors in their consultations explaining things to patients, asking about their overall health and well-being, and keeping an eye on them. As one woman who worked as a roadside seller said:

*Like I said before, nurses really have time for us and make us know there is nothing to be scared about. Because when you get scared the BP will go high so they normally have a chat with you in order for you to be well relaxed before your BP is taken.* (Patient interview 3)

Nurses agreed that they had an advantage over doctors as they could spend more time with patients and tailor their messages to help them understand their conditions. A nurse explained how patients’ comprehension improved:

*… when they come we take our time and explain things to them well. At the hospital you see the doctor who will not even ask you ‘how are you?’ when you enter because people are waiting. … They don’t want to waste time. The more time they spend on you, somebody will be annoyed somewhere so the person just looks into the folder and writes something for you … We take our time because we don’t have many clients here. We take our time and explain things to them so when they come here they are happy.* (Nurse interview 3)

Many nurses reported calling or showing up at the homes of patients who missed appointments. Participants shared examples of CVD nurses contacting patients either by phone or face-to-face if they did not show up for their scheduled appointments.

We heard few references to negative interactions between nurses and patients. One patient in a focus group stated that she did not feel looked after, and was disappointed that her CVD nurse had never called her to find out why she was not attending appointments. Another patient said that while his interactions with staff had been positive overall, one CVD nurse only laughed when he told her he experienced erectile dysfunction as a medication side effect, and did not alter his prescription or follow up.

Despite the overall positive assessment of the effectiveness of the programme, nurses shared their frustration at patients’ poor health seeking behaviour and missed appointments.

*Sometimes they don’t come. You call them. Some of their phones will be off. Some of them they said they can’t come and they will give you another date. This is the challenge.* (Nurse interview 2)

One nurse recounted a negative overall experience with ComHIP:

*For this community, nothing is working for me especially with my ComHIP. It got to a time I’ve been going to their house and when they see me then they start laughing. You will call and call and call, especially this community, I don’t know. They will not come, they won’t and they are not coming, they are not coming. They are not supportive of – I don’t know.* (Nurse interview 6)

##### Workload

All health professionals were asked about their workload, and while most said that it had increased with ComHIP, only two said they felt overloaded. One health professional in the hospital said that because she did not want to delay patients with long queues, she chose to do data entry for the programme after dinner in her own time. Most nurses said they did not find the added workload too much, and considered it to be part of the job.

The three healthcare professionals interviewed who worked at hospitals reported some frustration with increased workload. In particular, they complained about the more stringent screening regulations and adherence counselling brought by ComHIP. For example, the physician’s assistant said:

*With ComHIP we are supposed to check the BP three times at the consulting room with at least two minute intervals. It’s a challenge to us because at the consulting room where I work, at times we are only two clinicians seeing more than 200 clients. So imagine having even 50 hypertension clients – that means on average you are going to spend about ten minutes with each patient. So that’s a bit of a challenge for us*.

Conversations with the research assistants who conducted the interviews and local medical anthropologists suggested that nurses felt that this programme had increased their workload but not so much that they were overworked, however, ComHIP had increased their duties without extra remuneration.

##### Inability of nurses to dispense drugs

Many patients reported long waits in hospitals to get hypertensive medications and that by the time it was their turn, the supply would be depleted. It was often easier and quicker to pay out of pocket at private pharmacies. For some, getting to hospitals was time consuming and expensive, and few had their own means of transport to facilitate the journey.

Most community members expressed the opinion that anti-hypertensive drugs should be stocked at health centres. In the male FGD, when asked about their negative experiences, six of nine participants said the worst part of the programme was not obtaining their medications from CVD nurses.

*My challenge is that when we go to the nurses for check-ups we are referred to the hospital for medication, but I wish that the nurses would stock medicines so that after checking then I could have my medication. But considering my condition, if I have to go to the hospital I need transportation for two people and that becomes a problem, so I would want my medications to be given to me by the nurses*.

All nurses found it frustrating that they were unable to dispense anti-hypertensives, particularly when some patients could get drugs without a prescription illegally from unlicensed sellers. This was of particular frustration for midwives trained as CVD nurses as they had the ability to dispense other prescription drugs including antibiotics and HIV drugs, and they did not understand why they were not permitted to dispense anti-hypertensives.

#### Telemedicine consultations and referral of high risk patients to physicians

Most CVD nurses understood ComHIP referral procedures and referred high risk patients to doctors. However, one nurse reported referring all hypertensive patients to physicians, and another reported referring both moderate risk and high risk patients. While the system of referral appears to have worked well generally, these discrepancies reflect a shortcoming of the training, as not all nurses were aware of or followed appropriate referral guidelines.

Some participants stated that being enrolled in ComHIP granted them preferential treatment at clinics. One patient in the male FGD reported:

*What I will say is that this programme gives you the sense that when you go to the hospital, there is always somebody who is ready to listen to your problems. I remember there was a time when at the hospital people were many and the going was very tough for me … Previously when you went you didn’t know who to go to but right now you know that there is a particular team that you approach*.

Some health workers reported giving priority to ComHIP patients to keep their interest in the programme. Others said that they initially gave priority to ComHIP patients and then stopped because they wanted them to feel like "normal" patients. The debate over giving priority to ComHIP patients was a challenge to healthcare providers as they were worried that other non-ComHIP patients would feel that they were biased or being discriminated against.

Participants who had been referred but did not receive preferential treatment by doctors generally had more complaints and reported less personalised treatment and longer waiting times than participants under CVD nurses’ care. In addition, some patients reported issues with the referral process in which they were instructed to go to the hospital, but upon arrival received no designated care.

#### ICT messages for healthy lifestyles, treatment adherence and refill reminders for hypertensive patients

SMS were sent via the Voto platform. Participants were given a choice of text or voice messages, and could choose between English, Krobo, Twi or Ewe languages. Messages consisted of daily reminders to take medications; weekly health education messages; and reminders to fill prescriptions or schedule follow-up visits with health professionals. Participants had a choice of receiving their messages in the morning or evening. Initially, the programme required all participants to have a mobile phone, however, in the interests of inclusivity, this was changed to all participants having *access* to a mobile phone. This way, those who had access to a phone in their community or through relatives could join the programme.

When prompted, all patients agreed the SMS were appropriate, useful, and contained easy to understand messages, and they knew to talk to CVD nurses or doctors if they had difficulty understanding anything. When asked for specifics, most said the SMS helped them remember medicines, attend appointments, feel looked after, or empowered them to take control of their health. In a FGD, all participants said the SMS were helpful, with five of nine participants claiming that the SMS were the best part of the programme. Two women in a FGD reported that the SMS were vital to the programme, and said that without them they were not sure that they could take care of themselves or manage their hypertension.

All health professionals considered the SMS component to be excellent, and many found it to be one of the greatest strengths of the project. Nurses believed it particularly helped the patients remember to take their medications and return for their review appointments.

Despite the positive feedback about ComHIP’s SMS component, some challenges were apparent – mainly in terms of access. When asked about connectivity, there were conflicting responses. Some nurses replied that most patients had consistent access to network, but others reported that there were signal problems. Electricity was also a concern, and one nurse recounted that when a community member would go to the market, everyone would send along their phones for charging.

Many nurses expressed concerns about patients relying on a relative’s phone, particularly given they could miss messages when the relative was traveling. In our sample, only one participant said he did not have a phone and instead turned to the CVD nurses for information.

Some nurses suggested that in some instances too many SMS were sent, and that they were not useful to older participants. One nurse reported that a participant complained about receiving too many voice messages, but after switching to text messages he was satisfied.

One policy maker voiced concerns that the ICT portion would not be sustainable after FHI360 handed over to GHS during scale-up, as SMS delivery was centralised through CommCare, which was expensive. She suggested that it was possible that nurses could send them individually, as SMS were relatively cheap, but that would be the responsibility of the individual nurses.

#### Cloud based health records system linked to SMS messaging

The CommCare cloud based system was seen as one of the programme’s strengths, as all levels of health provider were able to access patients’ records at any time and track their management.

Health care professionals noted that the system made patients’ management easier and removed some decision-making. One pharmacist stated, *“With ComHIP it is easier; as soon as we monitor we will just key in and then you will receive a message as to what to do.”* Most nurses reported finding the tablet a useful tool as information systems. They used the tablets to search online for general health related information.

CommCare also faced challenges. As only the assigned nurse had access to each patient’s medical records, if a patient came in and his/her CVD nurse was not there, the available nurse was not able to access their records. Nurses could compensate by having other nurses write down their management of the patient, or by leaving their tablet or log in details so other nurses could access that patients’ data. However, problems occurred when doctors who were not part of the programme managed the patient without entering the appointment into the system. This led to visits and prescriptions not being registered, which also made it difficult to understand if patients were obtaining their medications or attending referral appointments. Some CVD nurses reported ongoing complexities in day-to-day use such as finding and entering codes, indicating that CommCare was not yet fluidly implemented for the nurses’ ease of use. Healthcare providers also reported that the CommCare platform was down for weeks at one point. Without a functioning network, or sufficient credit, it was impossible to enrol or manage patients according to ComHIP protocols.

Policy makers viewed CommCare as the most difficult component of ComHIP to scale-up. They suggested that the GHS would not be able to afford to provide tablets when these broke or disappeared, and without FHI360 support in the future this aspect would have to be done on paper, and SMS would not be sent automatically.

### Overall experiences of ComHIP

#### Health professionals’ experiences

With no exception, health professionals reported that the programme was better than previous hypertension measures in place. As previously nurses did not diagnose hypertension, there was no diagnosis at the community level unless a physician’s assistant was present. As one CVD nurse reported:

*Before ComHIP, we only checked the BP and when we saw that it was higher than normal we referred you to the clinician. From there, the client would come to us for anything again. BP was managed at the hospital until ComHIP came and then we also started managing BP patients. *(Nurse interview 7)

Nurses also reported that their equipment was much better post-ComHIP. Before, they only had one BP monitor per facility for everyone to share, making it impossible to screen during outreach as this would involve removing the only one from clinic. Now each centre had several, so they were able to screen in the community and at the clinic. As one clinician working in a hospital stated: *“I wish they could employ this method into the Ghana health system” *(Clinician interview) and the physician’s assistant said*: “I wouldn’t say it’s better; it’s the best! [Both laughing] It’s the best so far”.*

Several health professionals highlighted that ComHIP included more vigorous systematic screening with multiple readings thereby decreasing the risk of putting people at risk for unnecessary treatment. Some suggested that ComHIP reduced the load on hospitals, particularly for those who have money to pay for the drugs out of pocket. However, others reported that by following the new procedures there was an increased workload at hospitals, both due to boosted numbers of patients and stricter, time-consuming protocols.

#### Policy makers’ perspectives

Without exception, all policy makers familiar with ComHIP were cautiously supportive of the programme. Policy makers reported they believed the best way to combat hypertension was more prevention centred around health education and staff training, which were activities that ComHIP included. They felt that ComHIP could fill screening and health education gaps. When asked what the biggest strength of ComHIP was, a senior member of the GHS stated:

*The greatest strength of ComHIP is that it is a primary healthcare based intervention. If they had pitched it in a different way perhaps they would have had serious challenges but the primary healthcare base aligns with the Ghana Health Service, for that matter the Ministry of Health flagship intervention, and so it’s like you are doing something that is in the policy directive of the Ghana Health Service, and for that matter, the Ministry of Health.*

Policy makers interviewed familiar with ComHIP reported that they had been involved in the planning and design of the programme. Because a complete range of actors from senior policy makers to community members were included in discussions about the programme from the beginning, this added an additional level of ownership to the programme, which could help it succeed where other programmes had not. There was a feeling that FHI360 had listened to their concerns and priorities and the flexibility and adaptability of the programme was seen as one of its greatest strengths.

One place where communication broke down was with senior policy makers. Whilst FHI360 had involved the GHS in all decisions, due to staff turnover many of the people who were the contact points and responsible for ComHIP in the GHS had left, and their successors were not always appropriately informed of the programme. This caused frustration as they were being considered an active part of a programme that they had not heard of.

There was also a level of frustration at the district level. Health information obtained through CommCare went directly to FHI360 headquarters in Accra, where monthly reports on the health of their community members were produced and passed to the district. These reports were often delayed, and district officials expressed frustration about not receiving real time information that could have helped them to monitor their situation.

The sustainability of the programme also raised concerns. One informant thought it unlikely that the GHS, while having the capacity to train nurses in hypertension care, would be able to deliver tablets and other equipment without external support. A senior level health director expressed doubt that the GHS would be able to continue with CommCare, and was unsure if they would be able to continue the practice of automated SMS.

## Discussion

ComHIP is an attempt to bring integrated hypertension management into the community through a system of community education and nursing, referrals, and ICT support for participants. Everyone interviewed reported that ComHIP was a useful programme and a positive addition for hypertension management in this setting in eastern Ghana.

Patients reported finding the health education helpful and believed it had caused them to adopt healthy behaviours, though we heard of some nurses’ frustrations with patients’ health seeking behaviours. By and large, nurses reported that the programme had increased hypertension awareness among their patients, and they enjoyed their roles as educators. With the ComHIP training, the community nurses believed they were better placed than doctors to provide effective hypertension screening and management. However, we heard examples of nurses misinforming patients on care practices, indicating that ComHIP’s nurse training module could be improved.

Patients and nurses both reported that the programme had strengthened links and relationships between community members and nurses. Nurses said they felt more empowered and appreciated, while patients reported feeling better looked after.

Prior to ComHIP the only possibility for accessing hypertension care in Manya Krobo was in hospitals by physicians. Given the low doctor-to-patient ratio, it is imperative that innovative methods of reorganising care are utilised. Ghana currently has one doctor to 9043 patients [17]. This falls well below the 1/1320 patients per the recommendations of the WHO. The World Heart Federation’s hypertension roadmap recommends both task sharing and ICT as methods for control of hypertension in resource constrained settings [18]. Both of these techniques were fundamental to the structure of ComHIP.

### Task sharing

In areas where access to health services is constrained by a lack of health workers, the WHO recommends task sharing [19], which promotes lay and mid-level health workers in the provision of clinical tasks to offset the burden of a shortage of rarer, highly skilled professionals. Other programmes in sub-Saharan Africa have found at least limited success in the implementation of training nurses in the management of hypertension (for example in DRC [20]; in Nigeria [21]; in Kenya [22]; and Ghana [23]). Quantitative results show that while ComHIP succeeded in managing hypertension in patients who remained in the programme, there was a high loss to follow up [24]. Our qualitative analysis shows that had a positive response to the programme, suggesting that reasons other than its acceptability were the cause for this.

One unintended outcome of task sharing was a perceived increase in workload among CVD nurses and LCS, without a commensurate transfer of resources. Respondents had mixed opinions regarding ComHIP’s effect on their workload, with nurses indicating that while they were not overworked, ComHIP had increased their responsibilities without an increase in pay. While actual workload was not measured in this study, those working in hospitals, whilst admiring the increased rigour of the ComHIP screening process, reported that the programme was not sustainable given the large numbers of patients on their roster. For LCS, the anticipated rise in traffic to their shops was patchy, with many reporting that ComHIP had increased their responsibilities without remuneration. This feedback from the nurses and LCS involved with the programme suggests that some modifications to the programme be introduced. Possible short term measures include engaging LCS and helping them recognise the utility of capacity building associated with ComHIP; long term measures include capacity building of lower cadre GHS service providers to provide the services currently being provided by CVD nurses.

### Drug dispensing

The greatest frustration throughout ComHIP was access to medications, particularly the inability of LCS and CVD nurses to dispense medications. No patient in this study reported obtaining the wrong drugs or wrong doses, although CVD nurses anecdotally reported this was a common occurrence. CVD nurses found the inability to dispense medications frustrating, both because patients were not getting the correct medications in a timely fashion and because they did not understand why it was not possible. In many cases they were able to dispense medications for other conditions, and reported being able to obtain all HIV drugs for their community. Initially, drug distribution by CVD nurses was part of the ComHIP protocol, but the government did not grant permission. There are currently ongoing talks with the GHS to try and enable CVD nurses to, at least in some cases, dispense anti-hypertensives.

At least two projects in sub-Saharan Africa have shown promising results when allowing nurses to manage hypertension programmes and allowing nurses to provide anti-hypertensives. In one study in Kenya, nurses were allowed to prescribe drugs under Kenyan regulations [22]. This study found that nurses were able to successfully adhere to standard hypertension guidelines. However, this study did not include qualitative interviews with nurses or participants.

Another study conducted in Ghana identified a method of getting nurses to dispense anti-hypertensives. The task-shifting strategy for hypertension study (TASSH) conducted in the Ashanti region trained community nurses to deliver hypertension care [25]. This study showed a marked increase in nurses’ knowledge and practice of hypertension treatment. In this programme nurses were able to provide patients with anti-hypertensive medications for free. The nurses were able to circumvent the legal limitations by having the coordinating physician supply the needed drugs to the nurses. While this project was successful, it does not fit into the current regulations within the GHS that ComHIP is structured around, and may not be sustainable without a regular way for doctors to buy medications. Key to the conceptualisation of ComHIP is that it operates on already existing processes in the GHS, to improve its chances at sustainability once the programme ends.

Similar to our findings, patients in TASSH found the programme to be useful for increasing awareness of hypertension, and increasing their knowledge of behaviour and lifestyle options to increase heart health. However, the participants expressed concerns that as the medicines were provided to them, once the programme ended they would not be able to access medications. Community nurses involved in TASSH also spoke positively about the programme’s success in increasing hypertension awareness and knowledge of hypertension management, as well as the enjoyment of feeling they were making a difference, and having the opportunity to obtain more knowledge [25].

### Information and communication technology

The ICT portion of ComHIP was regarded as one of the strengths of ComHIP, and patients reported SMS to be useful for reminding them to take their medications and to teach them healthy behaviours. However, while there is great enthusiasm for SMS interventions for cardiovascular outcomes, there is still a lack of evidence that SMS campaigns are effective in changing health behaviours. A recent systematic review showed that while there was some evidence that SMS were effective for secondary prevention of cardiovascular disease, there is a lack of available evidence in low and middle income countries [26]. A further systematic review is currently underway to gather the available evidence on whether SMS programmes are useful for primary prevention of cardiovascular disease [27].

In addition, policy makers’ reservations on the sustainability of the SMS component in the Ghanaian context signals added attention must be given to securing support, including financial and political buy-in from local actors. More extensive ICT-based “digital medicine” approaches for the management of hypertension have been introduced in higher income countries with generally positive results [28–30]. Despite less robust infrastructure in LMICs such as Ghana, measures similar to those introduced by ComHIP should be implemented, tested and accompanied by sensitive evaluation to learn how to integrate them.

### Improved knowledge and the know-do gap

CVD nurses reported that ComHIP had increased hypertension awareness and knowledge in the community as well as their own personal levels of knowledge. LCS, community participants, and CVD nurses all reported that their knowledge of how to keep themselves healthy had increased during the programme.

It is well documented that knowledge is not enough to bring about behaviour change [31, 32], and ComHIP is designed to offer the opportunity for ease of access to medications and information about the disease as well as repetition and contact through its ICT component. By embedding itself in patients’ daily routines, the opportunity for medications to contribute to properly managing hypertension is greatly increased. Perhaps unsurprisingly then, nurses emphasised that there was still a great need for improved community health education, particularly on prevention and adherence to drugs, and that the GHS should take responsibility for more community health education.

Secondly, our results showed that despite the training and popular quarterly group exchange meetings, patients encountered (often unknowingly) nurses’ misrepresentation of clinical guidelines for hypertension management. These gaps in knowledge and misinformation on the part of the nurses underline the difficulty in translating new knowledge into accurate messages and appropriate behaviours. It also highlights the need for further careful and continual training of community nurses.

### Sustainability

The underlying motivation behind ComHIP was to build a project utilising the methods of the GHS, so that if successful, the project could be handed over to the GHS with little or no need for outside funding. Nevertheless, policy makers expressed some concerns about the long term sustainability of the programme. As it was based on the CHPS and utilised nurses, these parts were seen as being feasible in the long term. Our results suggest that by using training materials developed by FHI360 in conjunction with GHS, it is possible to train community nurses in hypertension management, although results of the effectiveness of the programme are pending. However, CommCare and the SMS components require outside resources that may be difficult to sustain, for example the tablets provided to the health staff. The ComHIP programme also requires greater numbers of BP monitors and scales than are commonly found in the community setting, and it is unclear who will pay for these in the long term.

### Strengths and limitations

The strength of this study is that it is a comprehensive study encompassing all major players and components of ComHIP. The main limitation is that while all efforts were made to ensure that participants covered were representative of the target population, it is possible that some groups may have been missed. There is possible courtesy bias on the part of all respondents, in that they may have told the interviewers what they thought they wanted to hear. Finally, a major limitation is that the study consisted solely of one-off interviews with little observation so the paper is largely built on participant reports.

## Conclusions and recommendations

The results of this study show that the greatest challenge this programme faced is the inability of CVD nurses to dispense anti-hypertensive drugs, despite being able to dispense drugs for other conditions in similar programmes. We suggest that all key stakeholders continue dialogue on the possibility of CVD nurses dispensing anti-hypertensive drugs.

Communication breakdown is a critical issue in resource poor settings, compounded by staff rotations and relocation. We therefore suggest similar programmes must continuously engage all stakeholders to ensure ongoing communication from beginning to end. Also, steps should be taken to ensure the sustainability of the programme, particularly the longevity of the technology component.

Finally, the findings of this study show how preliminary groundwork with actors at all levels involved in the roll out of this system needs to be undertaken before the implementation of any project, especially when related to sensitive subjects such as pharmaceuticals and the use of government trained or paid human resources.

## Data Availability

The dataset generated and/or analysed during the current study is available in the Harvard Dataverse repository, 10.7910/DVN/JJHOTB

## Abbreviations

CHPS: Community-based health planning and services
ComHIP: Community-based Hypertension Improvement Project
CVD: Cardiovascular disease
DBP: Diastolic blood pressure
FGD: Focus group discussions
GHS: Ghana Health Service
ICTs: Information and communication technologies
LCS: Licensed Chemical Sellers
LMICs: Low- and middle-income countries
NCDs: Non-communicable diseases
NHIS: National Health Insurance Scheme
PURE: Prospective Urban Rural Epidemiology
SBP: Systolic blood pressure
SMS: Short Message Service
TASSH: Task-shifting strategy for hypertension study
